# STAT3 inhibition for the induction of immunogenic cell death in multiple myeloma

**DOI:** 10.1080/2162402X.2026.2732727

**Published:** 2026-09-15

**Authors:** Oliver Kepp, Guido Kroemer

**Affiliations:** a INSERM US23 AMMICa, Institut Gustave Roussy, Université Paris-Saclay, Villejuif, France; b Inserm U1138, Centre de Recherche des Cordeliers, Equipe Labellisée par la Ligue Contre le Cancer, Université Paris Cité, Sorbonne Université, Paris, France; c Institut du Cancer Paris CARPEM, Department of Biology, Hôpital Européen Georges Pompidou, AP-HP, Paris, France

**Keywords:** Immunotherapy, napabucasin, calreticulin, integrated stress response

## Abstract

In a recent paper published in *Blood Science*, Sha et al. report that STAT3 inhibition eliminates multiple myeloma stem-like cells while eliciting hallmarks of immunogenic cell death. These highly intriguing findings connect stemness, integrated stress response, and antitumor immunity, yet in vivo validation remains necessary before this strategy can be considered immunogenic.

Multiple myeloma (MM) is therapeutically vulnerable yet rarely curable. Proteasome inhibitors, immunomodulatory drugs, antibodies, and cellular therapies can impose profound remissions, but relapse emerges from spatial heterogeneity, microenvironmental protection, and residual cells endowed with stem-like properties. Constitutive signal transducer and activator of transcription 3 (STAT3) signaling integrates interleukin-6-dependent survival, proliferation, immune escape, and stemness in MM, making it an attractive nodal target.^1^ ^,^ ^2^ The crucial question is whether STAT3 inhibition merely kills malignant plasma cells or converts their demise into an anticancer vaccine.

Sha and colleagues address this question with napabucasin (BBI608), an orally bioavailable compound commonly presented as a STAT3 inhibitor.^1^ Across human MM cell lines, napabucasin reduced viability, clonogenicity and STAT3 phosphorylation, induced G1 arrest and caspase-associated apoptosis, and retained activity in interleukin-6- or stromal-conditioned cultures. In KMS-11 cells, it contracted the Hoechst-excluding side population and suppressed LIN28A, NANOG, SOX2 and OCT4, suggesting specific effects on the stem cell-like compartment. Primary CD138-negative, CD19-positive, CD20-positive, and CD27-positive cells from 17 patients were more sensitive than CD34-positive umbilical cord blood cells. Finally, napabucasin prolonged survival of severely immunodeficient NOD-scid Il2rγ-null (NSG) mice (lacking functional B, T, and NK cells) bearing human RPMI 8226 MM without inducing measurable weight loss.^1^ Together, these observations suggest a therapeutic window, but neither molecular selectivity nor eradication of authentic relapse-initiating cells is yet demonstrated. Interestingly, Sha and colleagues observed that napabucasin synergized with two other drugs used for the treatment of MM, bortezomib and lenalidomide, in MM killing.^1^

The most original result is the connection between STAT3 blockade and immunogenic cell death (ICD). Transcriptomics revealed enrichment of the unfolded protein response (UPR) in MM cells treated with napabucasin. Immunoblotting documented phosphorylation of eukaryotic initiation factor alpha (eIF2α), which defines the integrated stress response (ISR), which is part of the UPR. eIF2α phosphorylation was accompanied by the activatory phosphorylation of its kinase EIF2AK3 (best known as PERK). Napabucasin-treated cells exposed calreticulin (CALR), HSP70, and HSP90 and released ATP. They also promoted CD80/CD86 upregulation on monocyte-derived dendritic cells and enhanced dendritic-cell-dependent T-cell cytotoxicity. A PERK inhibitor reduced this immune effect without reducing direct cytotoxicity, separating immunogenic signaling from cell killing.^1^ This is a coherent chain from stress sensing to immune activation and constitutes more than a mere collection of danger signals.

Several technical points regarding the implications of ISR deserve clarification. Napabucasin concentrations approaching 1 μM make pleiotropic off-target effects increasingly plausible. The PERK inhibitor experiment is suggestive, yet GSK2656127 was not shown to suppress eIF2α phosphorylation, CALR exposure, or ATP release. The authors suggested another eIF2α kinase, EIF2AK2 (best known as PKR) to be involved in napabucasin-elicited ICD, yet did not show any data in favor of this hypothesis.^1^ A STAT3 mutant resistant to inhibition, PKR knockdown, and PERK/PKR double perturbation would resolve these issues. Finally, ISR-dependent autophagic flux should be quantified, not inferred, using LC3 turnover and lysosomal blockade. Such experiments would determine whether the ISR is causal or merely accompanies cytotoxic stress.

Systematic screening established eIF2α phosphorylation as pathognomonic for ICD induced by clinically relevant agents.^3^ Mechanistically, this phosphorylation is required for both pre-apoptotic CALR exposure and autophagy, which enables premortem immunostimulatory ATP secretion.^4^ ^,^ ^5^ Two eIF2α kinases are especially relevant: PERK, activated by endoplasmic-reticulum stress, and PKR, which is activated by double-stranded RNA. Intriguingly, cytoplasmic STAT3 directly binds and inhibits PKR; STAT3 suppression consequently activates PKR, phosphorylates eIF2α, and unleashes autophagy.^6^ Napabucasin may therefore engage the ISR twice: through the measured PERK arm and through a plausible, but unmeasured, PKR arm (Figure 1). Testing PKR activation, autophagic flux, and their necessity would transform an attractive pathway diagram into a mechanism.

**Figure 1.**
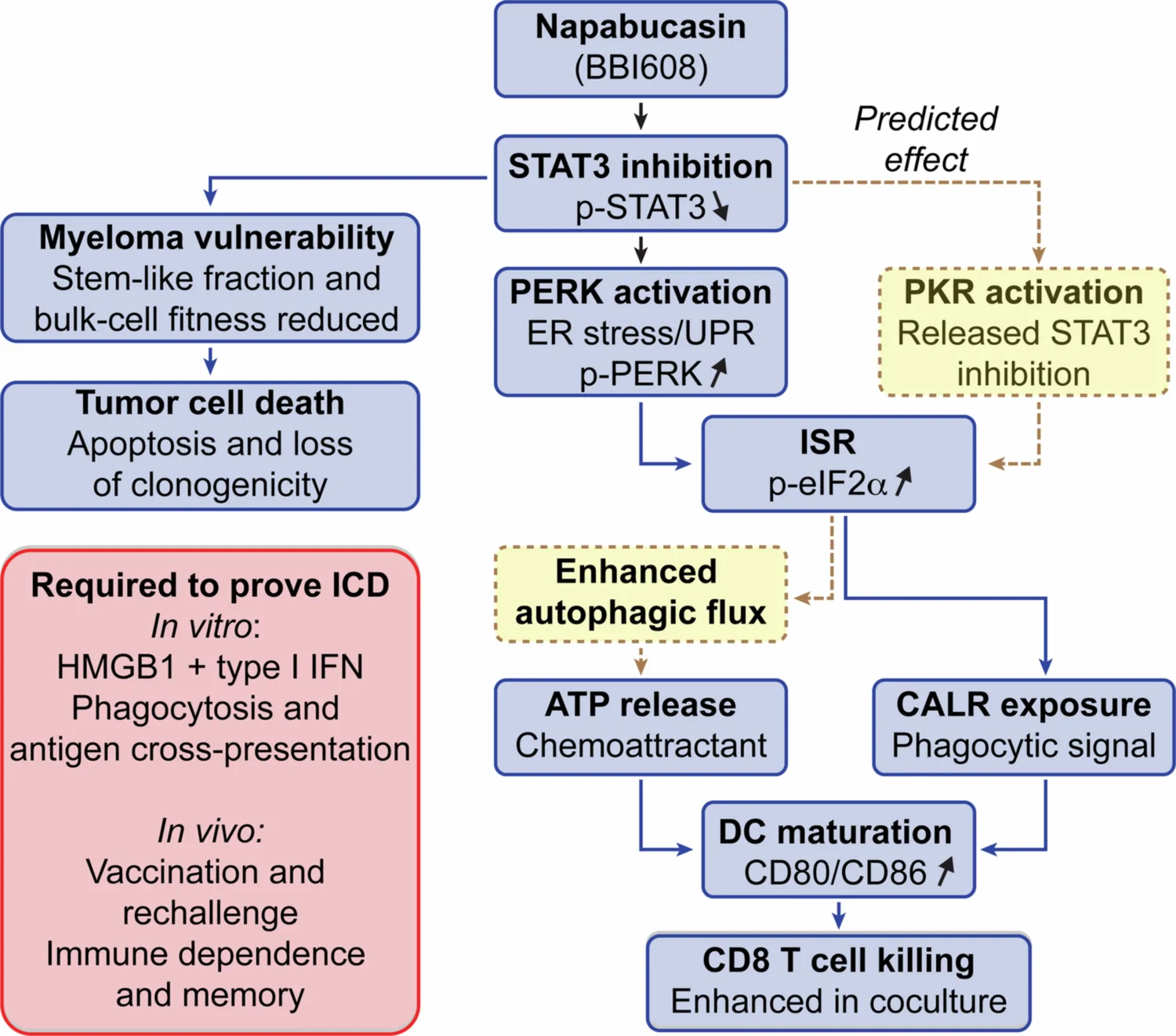
Proposed coupling of STAT3 inhibition to immunogenic cell death in multiple myeloma. Napabucasin inhibits STAT3 signaling, compromising bulk MM cells and the STAT3-dependent stem-like compartment. STAT3 inhibition may activate two convergent ISR branches: PERK activation, demonstrated by Sha et al., and release of PKR from direct STAT3-mediated inhibition, which remains to be tested in MM. PERK and PKR phosphorylate eIF2α, promoting autophagy-dependent ATP release and CALR exposure. These signals favor dendritic-cell activation and CD8-positive T-cell immunity. Blue boxes denote observations reported by Sha et al.; amber boxes denote mechanistically plausible but untested steps; red-outlined boxes identify decisive missing validation, including HMGB1/type-I-interferon measurements and vaccination/rechallenge experiments in immunocompetent mice. Abbreviations: CALR, calreticulin; DC, dendritic cell; eIF2α, eukaryotic translation initiation factor 2 alpha; ICD, immunogenic cell death; ISR, integrated stress response; MM, multiple myeloma; PERK, protein kinase R-like endoplasmic reticulum kinase; PKR, protein kinase R; STAT3, signal transducer and activator of transcription 3.

Does the study establish *bona fide* ICD? Not yet. Surface CALR and extracellular ATP are major hallmarks, but HMGB1 release, type-I-interferon production, CXCL10 secretion, phagocytosis, and antigen cross-presentation were not examined.^7^ ^,^ ^8^ HSP70/HSP90 exposure is supportive rather than defining. The dendritic-cell/T-cell cocultures provide functional reinforcement, although drug carryover, allogeneic responses, and undefined antigen specificity require control. Most importantly, ICD is an organismal property of dying cells, not a synonym for stress-associated DAMP emission. The gold-standard vaccination assay asks whether drug-killed cells protect immunocompetent, syngeneic mice against subsequent challenge with living tumor cells; therapeutic experiments should demonstrate immune dependence, durable memory, and rechallenge protection.^7^ NSG mice lack the adaptive immunity required for these tests. Thus, Sha et al. convincingly identify an ICD-compatible phenotype *in vitro*, but do not demonstrate ICD *in vivo*.

MM already offers two instructive precedents. Bortezomib exposes CALR, promotes dendritic cell phagocytosis, and elicits MM-specific immunity through cGAS-STING-dependent type-I-interferon signaling in immunocompetent models.^9^ Belantamab mafodotin, a BCMA-targeted antibody-drug conjugate, induces CALR exposure, ATP and HMGB1 release, dendritic-cell activation, CD8-dependent tumor control, immune memory, and antigen spreading.^10^ These examples establish both the biological plausibility and the evidentiary standard. Napabucasin should now be tested head-to-head with these agents, including combinations with bortezomib, for which Sha et al. report *in vitro* synergy but no immune-mechanistic dissection.

The translational promise lies in combining biological specificity with immunological amplification. STAT3-dependent stem-like MM cells may be preferentially vulnerable, allowing lower exposure and hence reduced toxicity, while ISR-driven ICD recruits host immunity to eliminate heterogeneous residual disease. Yet napabucasin cannot be assumed to be a selective STAT3 probe. Genetic STAT3 depletion and rescue, orthogonal inhibitors, pharmacodynamic target engagement, normal hematopoietic and immune-cell profiling, and dose-matched comparisons are indispensable. Immunocompetent or humanized MM models should then determine whether efficacy requires PERK, PKR, autophagy, CALR, dendritic cells, and CD8-positive T cells.

The conceptual proposition is compelling: disable a stemness circuit and simultaneously render the resulting cell corpses immunogenic. If validated rigorously, STAT3 inhibition could unite selective eradication of relapse-initiating MM cells with systemic immune control. The study therefore opens a promising door. Its next experiments must prove that immunity, rather than cytotoxicity alone, contributes to the therapeutic effects of napabucasin and other STAT3 inhibitors.

## Data Availability

This paper does not contain any original data.
